# Surgery and Actinomycin Improve Survival in Malignant Rhabdoid Tumor

**DOI:** 10.1155/2013/315170

**Published:** 2013-02-03

**Authors:** Ryan Horazdovsky, J. Carlos Manivel, Edward Y. Cheng

**Affiliations:** ^1^Department of Orthopaedic Surgery, University of Minnesota, 2450 Riverside Avenue South R200, Minneapolis, MN 55454, USA; ^2^Department of Laboratory Medicine and Pathology, University of Minnesota, 2450 Riverside Avenue South R200, Minneapolis, MN 55454, USA

## Abstract

*Purpose*. Malignant rhabdoid tumor (MRT) is an uncommon tumor that rarely occurs outside of renal and central nervous system (CNS) sites. Data from the literature were compiled to determine prognostic factors, including both demographic and treatment variables of malignant rhabdoid tumor, focusing on those tumors arising in extra-renal, extra-CNS (ER/EC MRT) sites. Patients and Methods. A systematic review and meta-analysis was performed by extracting demographic, treatment, and survival follow up on 167 cases of primary ER/EC MRT identified in the literature. *Results*. No survival differences were observed between those treated with or without radiation, or with or without chemotherapy. A Cox regression of overall survival revealed several independent prognostic factors. Surgical excision had a 74% (*P* = 0.0003) improvement in survival. Actinomycin had a 73% (*P* = 0.093) improvement in survival. Older age was associated with improved survival. The four-year survival, by Kaplan-Meier estimates, comparing patients less than two years old versus older than two at diagnosis was 11% versus 35%, respectively (*P* = 0.0001, Log-Rank). *Conclusion*. ER/EC MRT is a rare, soft-tissue tumor with a poor prognosis most commonly occurring in children. Surgical resection, treatment with actinomycin, and older age at diagnosis are all associated with improved survival.

## 1. Introduction

Malignant rhabdoid tumor (MRT) was first described as a rhabdomyosarcomatous subtype of Wilms Tumor in 1978 [1] and recognized as a distinct entity in 1981 [2]. Malignant rhabdoid tumors most commonly occur in children with extrarenal variants seen in the CNS, liver, female genital tract, and soft tissues. Extrarenal malignant rhabdoid tumor is a rare tumor with a poor prognosis. The medical literature is replete with papers describing the highly lethal nature of MRT. We report on demographics and treatment variables as prognostic after compiling data from the literature on extrarenal extra-CNS MRT. 

## 2. Methods

### 2.1. Eligibility and Search Strategy

A search was conducted for pure primary extrarenal extracentral nervous system malignant rhabdoid tumors. Ovid Medline was searched for articles containing “rhabdoid tumor” or listed under the MeSH “rhabdoid tumor” in English. This search was conducted between the years of 1981 and April 2006 as MRT was not recognized as a distinct entity until 1981. As this is a study of extrarenal extracentral nervous system MRT cases of paraspinal tumors were excluded. Cases described as carcinomas or other primary tumor types with “rhabdoid features” or a “rhabdoid component” were excluded. The inclusion criteria strategy was conducted with the understanding that there is not complete consensus on what constitutes a rhabdoid tumor. Our goal was to capture as many reports of malignant rhabdoid tumor as possible without including cases that could be deemed questionable. 

### 2.2. Data Abstraction

Data from 85 reports in the literature were compiled. Seven patients were dropped from analyses as they appeared to overlap between studies. This resulted in the identification of a total of 167 patients with extrarenal extracentral nervous system MRT. Follow-up data was available for 139 patients.

A database was constructed and available information was compiled including age at diagnosis, sex, site of primary tumor, time since diagnosis at the last followup, chemotherapy, individual chemotherapy drugs, radiation treatment, and surgery (either partial or complete resection). Life table analysis was performed in order to provide Kaplan-Meier estimates of survival after stratification separately for age and sex [3]. Differences in survival among subgroups were analyzed using a Cox proportional hazards analysis on other variables. Relative risks of death, ratio of mortality rates between subgroups, were estimated from the Cox regression model with and without adjustment for other factors [4].

## 3. Results

**Figure 1:**
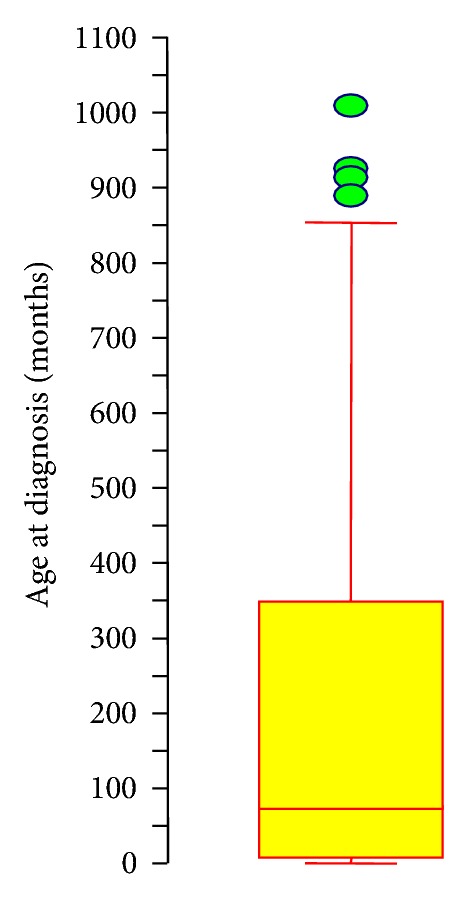
Age Boxplot. Boxplot showing median age (solid line within box) with borders of box representing 25th and 75th percentiles with 95% confidence intervals. Circles indicate individual outliers, the oldest of which is 1008 months at diagnosis.

Ninety-one patients were males and 76 patients were females giving a male to female ratio of 1.2. Age at diagnosis ranged from 0 weeks to 84 years old with a median age of 76 months (6 years) and a mean age of 197 months (16 years). As shown in Figure 1 the distribution of age at diagnosis was skewed towards younger patients. The mean time from diagnosis to the last followup was 19.3 months with a range of 0.25–192 months in the 139 patients where it was known. Overall survival at 4 years for the 139 patients with available follow-up data was 0.245 ± 0.083 (95% confidence interval). Of the 75 males with available follow-up data, overall survival at 4 years was 0.196 ± 0.112 (95% confidence interval). Of the 62 females with followup, overall survival at 4 years was 0.304 ± 0.128 (95% confidence interval). Survival between males and females was not statistically different (*P* = 0.36 Wilcoxon, *P* = 0.26 Log-rank). Refs for table from MRT search on March 13, 2006 [5–85].

### 3.1. Analysis of Age at Diagnosis and Survival

**Figure 2:**
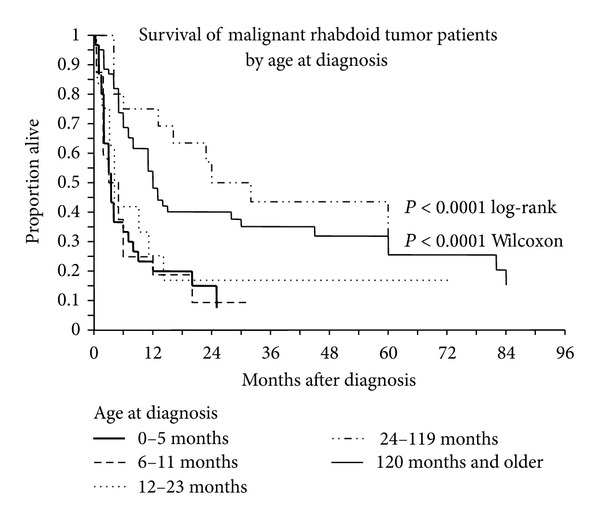
Analysis of age at diagnosis and survival. Kaplan-Meier survival curves of patients diagnosed with extrarenal extra-CNS malignant rhabdoid tumor. Age classes under 2 years old were indistinguishable. Age class 2–10 years had the best prognosis and were significantly different than those age classes under 2, but were not different from the >10-year-old age group. The >10 age group was significantly different from the <2-year-old group.

**Figure 3:**
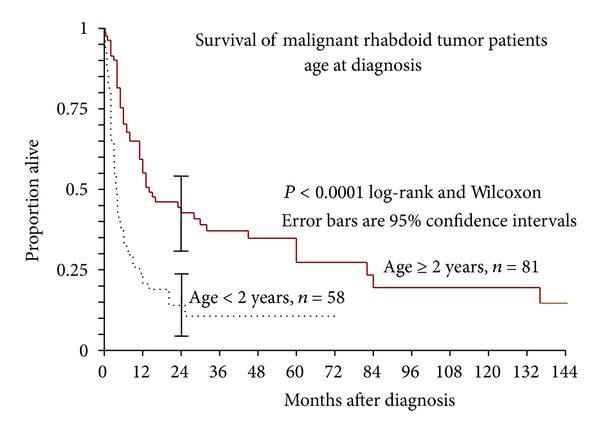
Kaplan-Meier survival of patients diagnosed with extrarenal extra-CNS malignant rhabdoid tumor with four-year survival comparing patients less than two years old versus older than two at diagnosis was 11% versus 35%, respectively (*P* = 0.0001).

Patients were stratified into 5 age classes based on age at diagnosis: Class 1 (0–5 months), Class 2 (6–11 months), Class 3 (13–23 months), Class 4 (24–119 months or 2–10 years), and Class 5 (>120 months or 10 years) (Figure 2). These age classes were chosen to coincide with a recent report on renal malignant rhabdoid tumor and illustrate differences in survival based on age [86]. The age classes under 2 years (Classes 1, 2, and 3) were indistinguishable. The 2–10-year-old group (Class 4) had the best prognosis and was significantly different from the combined Classes 1, 2, and 3 (*P* = 0.0001 Wilcoxon). The 2–10-year-old group (Class 4) appeared to have a better prognosis than the >10-year-old group (Class 5), but this was not significant (*P* = 0.18 Wilcoxon). The >10-year-old group was different from the 0–2-year-old group (*P* < 0.0001 Wilcoxon). Based on the above results, the ages at diagnosis were lumped into two groups: less than 2 years old and greater than or equal to 2 years. Overall survival in these two groups was significantly different (*P* < 0.0001 Wilcoxon) (Figure 3), with survival statistics as seen in Table 1.

Of 50 deaths in the <2 group, only one occurred at more than two years after diagnosis (25 months). Of 53 deaths in the older group, 8 occurred after two years, with the last at 11.3 years.

### 3.2. Analysis of Treatment and Survival

**Figure 4:**
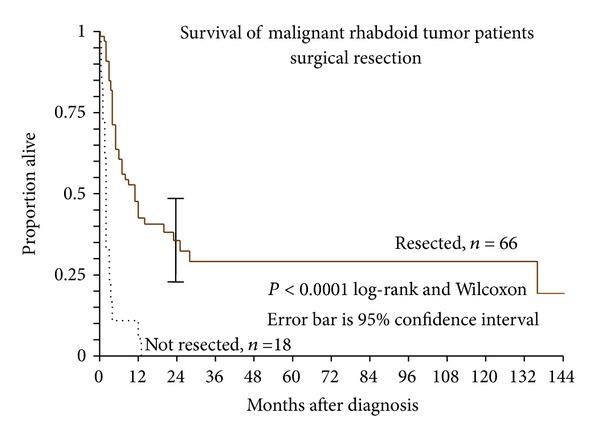
Kaplan-Meier survival of malignant rhabdoid tumor patients with and without surgical resection.

Sixty-nine patients with available followup were reported as undergoing surgical resection (partial or complete) while 18 patients did not undergo surgery (Figure 4). Most of the articles did not specify whether surgical resections were partial or complete. Therefore, we compared patients who had not had surgical resection to patients who had any type of surgical resection (partial or complete). Surgical resection was a highly significant factor reducing the risk of death by 74% (*P* = 0.0003; hazard ratio 0.26, 95% C.I. 0.12 to 0.55). Most of the patients that did not have surgery were reported as having an unresectable tumor.

Resected patients were significantly older at diagnosis than those who went unresected (*P* = 0.0027 by Mann-Whitney “*U*” test). Median age at diagnosis for resected patients was 168 months (14.0 years); for nonresected patients this figure was 5 months (0.42 years). While this marked age difference could be due to differences in resectability of a tumor in an older child versus a neonate, unfortunately, the lack of data regarding actual resectability, as opposed to whether or not a surgical resection was done, precluded a more in-depth analysis of this potential issue. Despite their association, age at diagnosis and resection separately had a significant impact on overall survival (Table 2).

**Figure 5:**
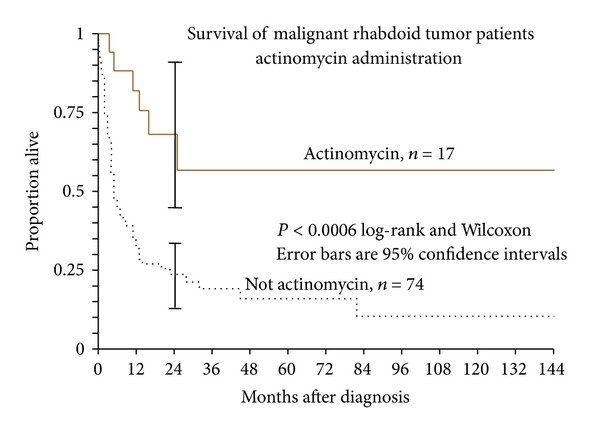
Kaplan-Meier survival estimates of patients treated with and without actinomycin. Actinomycin is associated with a 74% reduced risk of death (*P* = 0.0059) which held true for all age groups.

 Ninety-six patients with available followup received some form of chemotherapy while 15 did not. Eighty-nine of the patients receiving chemotherapy had a multidrug regimen, but the regimens were not consistent. There was no statistically significant survival benefit with or without chemotherapy as a whole (*P* = 0.75). However, inclusion of actinomycin in a multidrug regimen was associated with a 73% reduced risk of death (*P* = 0.0093, hazard ratio 0.28, Table 2), which held true for all age groups. Seventeen patients with available followup received actinomycin while 77 patients did not (Figure 5). A univariate analysis suggested that the regimens containing actinomycin had a longer survival and this was independently significant upon Cox regression. While this does not demonstrate a superior efficacy of actinomycin in this tumor, it suggests that actinomycin should be included in multidrug chemoregimens for this tumor. 

Fifty-nine patients with available followup were reported as receiving doxorubicin as part of their multidrug regimen, while 35 patients were reported as not receiving doxorubicin. Inclusion of doxorubicin in a multidrug regimen did not have a significant impact on survival (*P* = 0.84). Seventeen patients were reported as receiving cisplatin while 78 patients were reported as not having received cisplatin. Inclusion of cisplatin in a multidrug regimen was associated with a 111% increased risk of death (*P* = 0.0466). The increase in risk associated with cisplatin was analyzed further by looking at associations among cofactors. Age at diagnosis and cisplatin had a weak association, (*P* = 0.07 by Mann-Whitney “*U*” test). Median age of diagnosis for patients taking cisplatin was 12 months (1.0 year), while for those not taking cisplatin the median age at diagnosis was 81.6 months (6.8 years). Resection and cisplatin also had a weak association (2 × 2 table, Fisher's exact probability, *P* = 0.067) with cisplatin associated with the unresected patients. From the associations between age at diagnosis, resection, and cisplatin, it appears that younger patients were receiving cisplatin, but that their disease severity rendered it ineffective.

Forty-five patients were reported to have received radiation therapy while 66 were reported as not having received RT. There was no statistically significant effect of treatment with or without RT (*P* = 0.6705). Table 2 displays a Cox regression of six of the cofactors discussed.

### 3.3. Analysis of Tumors of Soft Tissues

In this analysis tumors of the ovary (1 case), bladder (2), colon (2), small intestine (2), esophagus (1), liver (16), prostate (3), lungs (2), thymus (1), thyroid (1), stomach (1), uterus (5), heart (1), and lacrimal gland (1) were excluded. Tumors of the soft tissue showed a weak trend toward improved survival with a decreased risk of death of 40% (*P* = 0.11; hazard ratio 0.605, 95% C.I. 0.321 to 1.14). There was no difference between the “soft tissue” group and excluded group in regards to the report of a surgical resection (*P* = 0.27). The analysis of the soft tissue subgroup showed the same trends in regards to age at diagnosis as the ER/EC MRT group as a whole. 

Further analysis of the “soft tissue” group showed 7 sites with more than 3 reported cases: chest wall (11 cases), inguinal (5), neck (15), orbit (4), pelvis (8), shoulder (8), and vulva (7). Chi-squared and Cox regression analysis showed no evidence that site in these subgroups had any effect on rate of resection or survival. 

Finally, we examined subgroups of cases that included 15 in the neck, 32 in the extremities (20 proximal and 12 distal), and 67 involving the trunk (one of them also in the neck and one also in the thigh). Statistic chi-squared showed no difference in rates of resection between these sites. A Cox regression of these subgroups showed no difference in survival.

## 4. Discussion

Reports in the literature have shown poor long-term survival rates in patients with extrarenal extra-CNS MRT. Particularly troubling are the dismal survival rates for the very young. Our reported overall survival for patients diagnosed at less than two years of age of 11% is close to the 9.1% survival for fetal and neonate ER/EC MRT reported by Isaacs [87]. Tomlinson et al. demonstrated a strong correlation of increasing survival with increasing age at diagnosis for patients with renal malignant rhabdoid tumors which is similar to what we have found for ER/EC MRT [86]. Similar to our study, a German review of 70 pediatric cases of malignant rhabdoid tumor of varying locations showed no improvement in outcome with radiotherapy [88]. 

Absence of INI1 is valuable in confirming the diagnosis of renal or extrarenal MRT versus other tumors with focal rhabdoid appearance [89]. INI1, associated with chromatin remodeling and expressed in all tissues, is a product of the hSNF5/INI1 tumor suppressor gene which has been demonstrated to be frequently mutated or deleted in MRTs [90, 91]. Cytogenetic study of malignant rhabdoid tumors (renal and extrarenal) has shown a region of common deletion at 22q11. Analysis of chromosome 22q has been used as an aid to the diagnosis of rhabdoid tumors [24]. A putative tumor suppressor gene locus, hSNF5/INI1, has been identified. Mitotic recombination, nondisjunction, duplication, or deletion in the proximal part of chromosome 22q appears to be associated with hSNF5/INI1 inactivation [92]. Reexpression of the *hSNF5* gene in MRT cell lines induces G1 cell cycle arrest and activation of senescence-associated proteins [93–96].

Genetic alterations in chromatin remodeling complexes appear to be responsible for the formation of cytoplasmic perinuclear inclusion bodies seen in MRT. The cytokeratin (CK) 8 gene analyzed from human MRT showed missense mutations in genes whose products are involved in lateral protofilament-protofilament interactions and phosphorylation sites important to filament organization [97]. Alterations to regions involved in microfilament conformational change appear to interfere with chromatin remodeling in MRT.

Current understanding of the genetic similarities observed in rhabdoid tumors would support establishing common treatment protocols; however, the relative rarity of the disease has made this difficult. Resections to the extent possible, chemotherapy, and radiation therapy are often employed together in the treatment of this disease. Chemotherapy regimes for treatment of MRT have included various combinations of cisplatinum, cyclophosphamide, adriamycin, and VP-16 [20, 66, 98]. There are reports of using the German Society of Pediatric Oncology Protocol (HIT, procarbazine, ifosfamide, VP-16, methotrexate, cytosine-arabinoside, and cisplatin) [98]. With no well-established adjunct treatment protocols our data suggests that an aggressive surgical excision is indicated in cases of operable MRT. In addition, our retrospective review and analysis of the literature would support the consideration of a chemotherapy regime that includes treatment with actinomycin.

## Figures and Tables

**Table 1:** Kaplan-Meier survival estimates showing the proportion of patients surviving at given time from diagnosis.

Age at diagnosis	<2 years	≥2 years
*N *	58	81
Deaths	50	53
2-Year K-M Estimate	0.1422	0.4773
95% CI.	0.0476 to 02369	0.3121 to 0.5424
5-Year K-M Estimate	0.1067	0.2737
95% CI.	0.0135 to 0.1999	0.1548 to 0.3925

**Table 2:** Results of Cox regression of overall survival on six cofactors.

Cofactor	*P* value	Hazard Ratio
Resection (Y/N)	0.0003	0.26
Actinomycin (Y/N)	0.0093	0.28
Age ≥ 2 years (Y/N)	0.0140	0.43
Male (Y/N)	0.46	1.26
Radiation (Y/N)	0.94	0.97
Chemotherapy (Y/N)	0.99	1.01
